# Grading variation in 2,934 patients with ductal carcinoma in situ of the breast: the effect of laboratory- and pathologist-specific feedback reports

**DOI:** 10.1186/s13000-020-00970-8

**Published:** 2020-05-11

**Authors:** Carmen van Dooijeweert, Paul J. van Diest, Inge O. Baas, Elsken van der Wall, Ivette A. G. Deckers

**Affiliations:** 1 Department of Pathology, University Medical Center Utrecht, PO Box 85500, 3508 GA Utrecht, the Netherlands; 2 Department of Medical Oncology, University Medical Center Utrecht, Utrecht, the Netherlands; 3 Foundation PALGA (the nationwide network and registry of histo- and cytopathology in the Netherlands), Houten, the Netherlands

**Keywords:** DCIS, ductal carcinoma in situ, histologic grade, patient management, laboratory-specific feedback, daily pathology practice

## Abstract

**Background:**

Histologic grade of ductal carcinoma in situ of the breast (DCIS) may become the single biomarker that decides whether patients will be treated. Yet, evidence shows that grading variation in daily practice is substantial. To facilitate quality improvement, feedback reports, in which laboratory-specific case-mix adjusted proportions per grade were benchmarked against other laboratories, were sent to the individual laboratories by March 1, 2018. One year later, the effect of these feedback reports on inter-laboratory variation was studied.

**Methods:**

Synoptic pathology reports of all pure DCIS resection specimens between March 1, 2017 and March 1, 2019 were retrieved from PALGA (the nationwide Dutch pathology registry). Laboratory-specific proportions per grade were compared to the overall proportion in the year before and after feedback. The absolute deviation for all three grades at once, represented by the overall deviation score (ODS), was calculated as the sum of deviations from the grade-specific overall proportions. Case-mix adjusted, laboratory-specific odds ratios (ORs) for high- (grade III) versus low-grade (grade I-II) DCIS were obtained by multivariable logistic regression.

**Results:**

Overall, 2954 DCIS reports from 31 laboratories were included. After feedback, the range between laboratories decreased by 22 and 6.5% for grades II and III, while an increase of 6.2% was observed for grade I. Both the mean ODS (27.2 to 24.1%) and maximum ODS (87.7 to 59.6%) decreased considerably. However, the range of case-mix adjusted ORs remained fairly stable and substantial (0.39 (95% CI: 0.18–0.86) to 3.69 (95% CI: 1.30–10.51)).

**Conclusion:**

A promising decrease in grading variation was observed after laboratory-specific feedback for DCIS grades II-III, while this was not observed for DCIS grade I. Overall, grading variation remained substantial which needs to be addressed considering its clinical implications. Nationwide consensus on a classification, and training of (expert breast) pathologists, for example by e-learning, may help to further improve grading standardization.

## Introduction

Treatment of ductal carcinoma in situ of the breast (DCIS) currently consists of surgery [1, 2], radiotherapy [2–6] and sometimes even (low-dose) tamoxifen [2–4, 7–9]. However, it is believed that an unknown number of DCIS patients are treated for lesions that may never progress into invasive breast cancer [10–13]. Therefore, four randomized controlled clinical trials aim to identify a group of low-risk DCIS patients that, under active surveillance, may safely forgo surgical treatment [11, 12, 14–16].

For all trials, the main biomarker which identifies DCIS as being low-risk, is histologic (nuclear) grade, although different classifications are used by the LORD-, LORIS-, COMET-, and LARRIKIN trials [11, 12, 14–16]. The general hypothesis of these trials is that progression risk, or at least speed of progression is higher for high-grade lesions [17, 18], and if a low-grade DCIS does become invasive, it will be a low-grade invasive carcinoma with favorable characteristics and excellent survival rates after treatment [11, 19].

Besides the fact that grade may become the single biomarker that is used to decide whether patients are treated for their DCIS, grade already plays an important role in clinical patient management. For example, grade influences radiotherapy decisions (omitting a boost, considering partial breast irradiation) [1, 6] and indicates (on biopsy) whether a sentinel lymph node procedure is required [1]. Thus, accurate, consistent, and reproducible grading is of key importance. However, we previously showed that variation in grading, between pathology laboratories and between pathologists within laboratories, is substantial in daily clinical practice on a nationwide scale in the Netherlands [20]. Furthermore, studies in which a set of DCIS was assessed by several pathologists showed significant inter-observer variation, regardless of the used classification, as well [21–23].

As studies have shown that quality of breast cancer care can be improved by auditing and benchmarking [24–29], the results of our previous study were sent to all participating Dutch pathology laboratories as feedback reports, in which their proportions per grade were benchmarked against other laboratories. This enabled pathologists to discuss and reflect upon their grading practices. The present study was conducted to investigate the effect of these feedback reports on grading variation between laboratories on a nationwide scale.

## Methods

### Data source

All data were retrieved from PALGA, the Dutch nationwide network and registry of histo- and cytopathology, which contains excerpts of all pathology reports from laboratories in the Netherlands [30]. Data within this database are pseudonymized in the laboratories themselves and by a trusted third party (ZorgTTP, Houten, the Netherlands). In addition, as data on the reporting pathologist was not available in PALGA, laboratories provided the pathologist information directly to the PALGA data-analyst. In a final step, the PALGA data-analyst anonymized all laboratories and pathologists to the researchers. This study was approved by the scientific and privacy committee of PALGA and data were retrieved and handled in compliance with the General Data Protection Regulation Act (GDPR).

### Study population

We retrieved all synoptic pathology reports of patients with pure DCIS resection specimens (i.e. without a report of a known adjacent invasive breast cancer) in the Netherlands between March 1, 2017 and March 1, 2019 from PALGA (*n* = 3336). Per pathology report, patient- and tumor characteristics were extracted (sex, age, type of surgery, histologic grade and tumor size). Reports with missing data on histologic grade and/or tumor size were excluded (Supplementary Fig.1). Pathology reports of residual in situ lesions after neoadjuvant treatment of primary invasive breast carcinomas were excluded. Furthermore, ipsilateral DCIS reports within six months of the previous DCIS resection specimen report were considered paired measurements of which we only included the first report.

In total, 38 out of 42 pathology laboratories in the Netherlands reported resection specimens via the synoptic (PALGA) protocol from March 1, 2017 and onwards. Of these 38 laboratories, we included those that annually reported a minimum of 15 DCIS resection specimens within the protocol.

### Feedback reports

Laboratory-specific feedback reports, with proportions per histologic grade of individual laboratories benchmarked against other anonymized laboratories [20], were sent out by March 1, 2018. The general feedback report is available on the PALGA website (in Dutch only) [31], while laboratory-specific reports are only available to the individual laboratories themselves.

Laboratory-specific reports consisted of funnel plots, in which absolute differences in proportion of histologic grade, are plotted against the number of included IBC per laboratory. Within these funnel plots, the national proportions per grade with their 95% confidence intervals as limits were set as targets.

In addition, all laboratories were asked to provide coded information of the reporting pathologist per pathology report, to compare grading practices of the different pathologists within their laboratory. These data on pathologist’ level were provided by ten laboratories, which as a result received feedback on both laboratory- and pathologist’ level. Thus, pathologists within these ten laboratories were enabled to discuss and reflect upon both their personal- and overall laboratory grading practices.

As feedback reports were sent to the laboratories by March 1, 2018, we considered the period from March 1, 2017 up to and including March 1, 2018 as pre-feedback period, while the period from March 2, 2018 up to and including March 1, 2019 is considered the post-feedback period.

### DCIS grading classification

Histologic grade was defined in the pathology reports as grade I, II or III, without a specification of which classification was used. The Dutch guideline [1] recommends to use the classification of Holland [32]. However, we know from our previous research that numerous different guidelines are used by Dutch pathologists in daily practice [20].

### Statistical analysis

Patient and tumor characteristics from the pathology reports were summarized in Table 1. Differences of these characteristics between pre- and post-feedback pathology reports were tested by means of a χ^2^-test for categorical variables and by means of a Mann-Whitney U test for continuous variables.

**Table 1:** Characteristics of the 2954 included ductal carcinoma in situ (DCIS) lesions from the PALGA database between March 1, 2017 and March 1, 2018 (PRE feedback), and March 1, 2018 to March 1, 2019 (POST feedback)

	Total (*n* = 2954)	PRE (*n* = 1493)	POST (*n* = 1461)	***p***-value^**b**^
Histologic grade, *n* (%)				
Grade 1	383 (13.0%)	169 (11.3%)	214 (14.6%)	**0.016**
Grade 2	1171 (39.6%)	590 (39.5%)	581 (39.8%)	
Grade 3	1400 (47.4%)	734 (49.2%)	666 (45.6%)	
Age (y)^a^	59.8 (10.3)	59.8 (10.5)	59.8 (10.1)	0.922
Sex, n (%)				0.790
Female	2943 (99.6%)	1487 (99.6%)	1456 (99.7%)	
Male	11 (0.4%)	6 (0.4%)	5 (0.3%)	
Tumor size (cm)^a^	2.5 (2.3)	2.5 (2.3)	2.5 (2.3)	0.321
Type of surgery, n (%)				0.096
Mastectomy	944 (32.0%)	456 (30.5%)	488 (33.4%)	
Breast conserving	2010 (68.0%)	1037 (69.5%)	973 (66.6%)	

^a^ Mean (SD)

^b^ PRE vs POST categorical variables by Chi-square test, continuous variables by Mann Whitney U Test

Overall proportions for DCIS grades I-III were determined pre- and post-feedback. Absolute differences from the overall proportions per laboratory are presented in bar charts per feedback period for grades I-III. We calculated an overall deviation score (ODS), consisting of the sum of absolute deviations from the grade-specific overall proportions per period, to compare the absolute deviation for all grades at once. Differences in ODS of the individual laboratories between the pre- and post-feedback period were compared by means of a Wilcoxon signed-rank test.

We used several, arbitrary, definitions of change as a possible way to interpret the type of change in laboratories. Laboratories who showed an absolute change of ≤2% were defined as ‘not shifting’. Among laboratories that showed an absolute change of > 2%, laboratories moving closer to the overall mean after feedback were defined as ‘less deviant’, while laboratories moving further from the overall mean after feedback were defined as ‘more deviant’.

A logistic regression analyses, providing odds ratios (ORs) and 95% confidence intervals (CI) per laboratory, was used to compare relative differences among laboratories. For the logistic regression model, grade was dichotomized into low-grade DCIS (grade I-II) and high-grade DCIS (grade III), as this is the same definition that is used by the majority of the clinical trials [11, 12, 14, 16, 20]. The laboratory best resembling the national distribution on low- (grade I-II) and high-grade (grade III) was chosen as reference laboratory. We performed a multivariate logistic regression analysis to correct for differences in case-mix. Variables were selected based on our previous research [20] and consisted of tumor size and type of surgery.

To compare differences in case-mix adjusted ORs of the individual laboratories, the positive OR difference, i.e. the difference of the laboratory OR to the reference OR of 1.00, was calculated. These positive OR differences of the individual laboratories were compared pre- and post-feedback by Wilcoxon signed rank test.

All analyses were performed by using IBM SPSS Statistics version 15.0.0.2.

## Results

### Patient-, tumor- and laboratory characteristics

In total, 2954 DCIS resection specimen reports from 2934 patients were included. For 20 patients, two pathology reports were included, as this either concerned a bilateral tumor (*n* = 18) or an ipsilateral tumor > 6 months after the first diagnosis (*n* = 2). Of the included reports, 1493 were defined as pre-feedback, while 1461 were defined as post-feedback.

Characteristics of the included DCIS resection specimen reports are summarized in Table 1. All included pathology reports originate from 31/42 pathology laboratories. Four laboratories did not implement synoptic reporting between March 1, 2017 and March 1, 2019. The remaining seven laboratories were excluded as they synoptically graded less than 15 DCIS lesions within the PALGA protocol pre- and/or post-feedback. The overall number of DCIS reports before feedback ranged from 19 to 94 (median 48), whereas after feedback the number of synoptic pathology reports ranged from 19 to 86 (median 39).

Mean age (SD) at diagnosis was 59.8 (10.3) years and mean tumor size (SD) was 2.5 (2.3) cm. Overall, only 11 men were included (0.4%). Breast conserving surgery was performed in almost 70% of DCIS patients, although a small decrease was observed after feedback (69.5% versus 66.6%, *p* = 0.096). A significant change in distribution of histologic grade was observed after feedback (*p* = 0.016), as the proportion of grade I increased (from 11.3 to 14.6%), whereas the proportion of grade III decreased (from 49.2 to 45.6%), while grade II remained fairly stable (39.5% versus 39.8%) (Table 1).

### Inter-laboratory differences in histologic grading

**Fig. 1:**
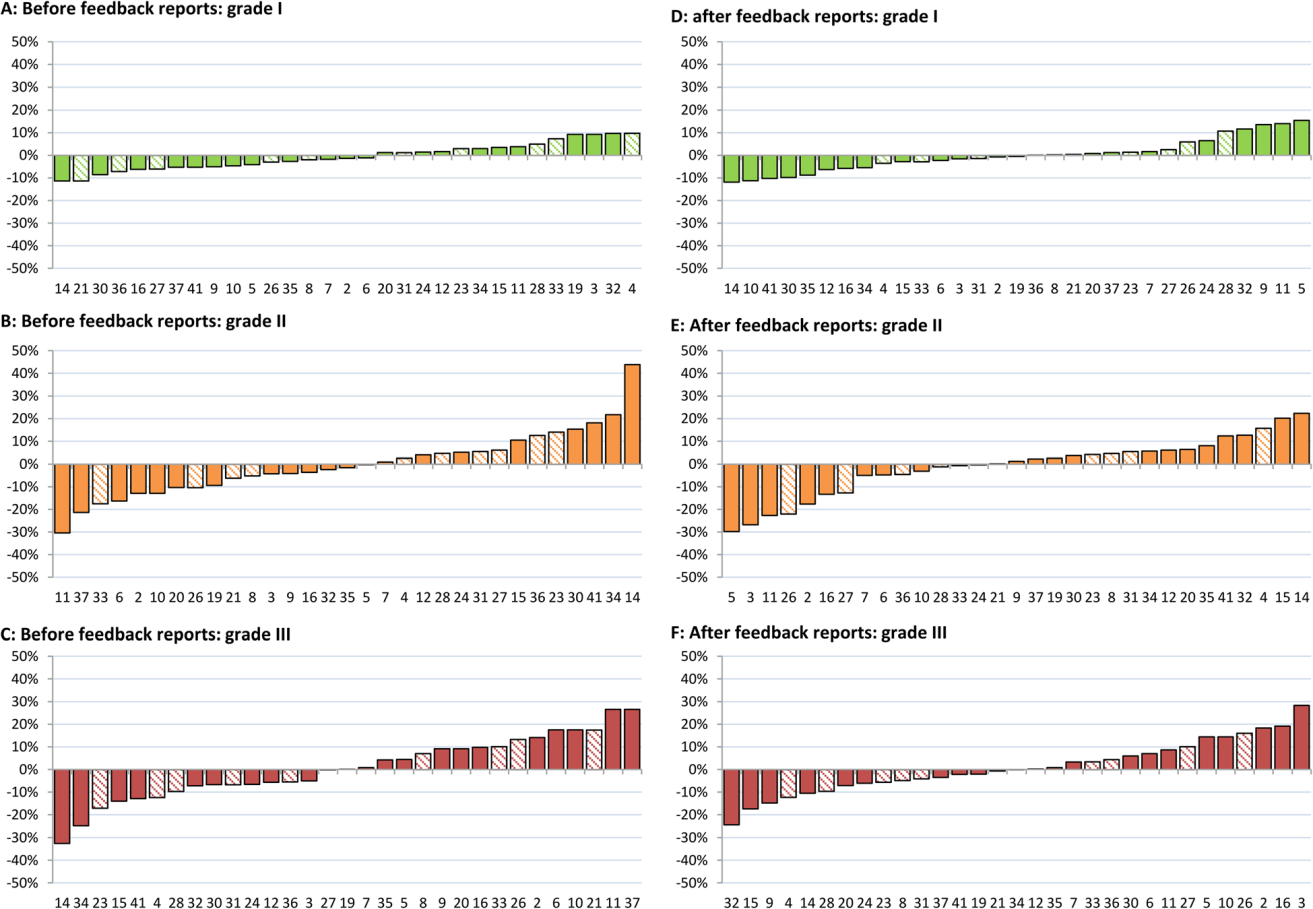
Inter-laboratory (*n* = 31) variation in histologic grading of ductal carcinoma in situ (DCIS) of the breast before (**a-c**) (*n* = 1493) and after feedback reports (**d-f**) (*n* = 1461). Percentages per laboratory show the absolute deviation from the national proportion per histologic grade for grade I (A + D), grade II (B + E) and grade III (C + F). Laboratory numbers for all sub-figures (**a-f**) correspond. All laboratories are ranged from lower (negative values) to higher proportions (positive values). A decrease in absolute range was observed for grades II and III; grade II (−22.0%), grade III (−6.2%). An increase was observed for grade I (+ 6.5%). Striped bars indicate laboratories who received feedback on pathologist’ level (*n* = 10)

For grade II, the total range between laboratories decreased markedly by 22% (from 9.1–83.3% to 10.0–62.2%) and, for grade III, to a lesser extent by 6.5% (from 16.7–75.8% to 21.3–73.9%) (Fig. 1). In contrast, the range between laboratories increased by 6.2% for grade I (from 0.0–21.1% to 2.7–30.0%) (Fig. 1).

**Fig. 2:**
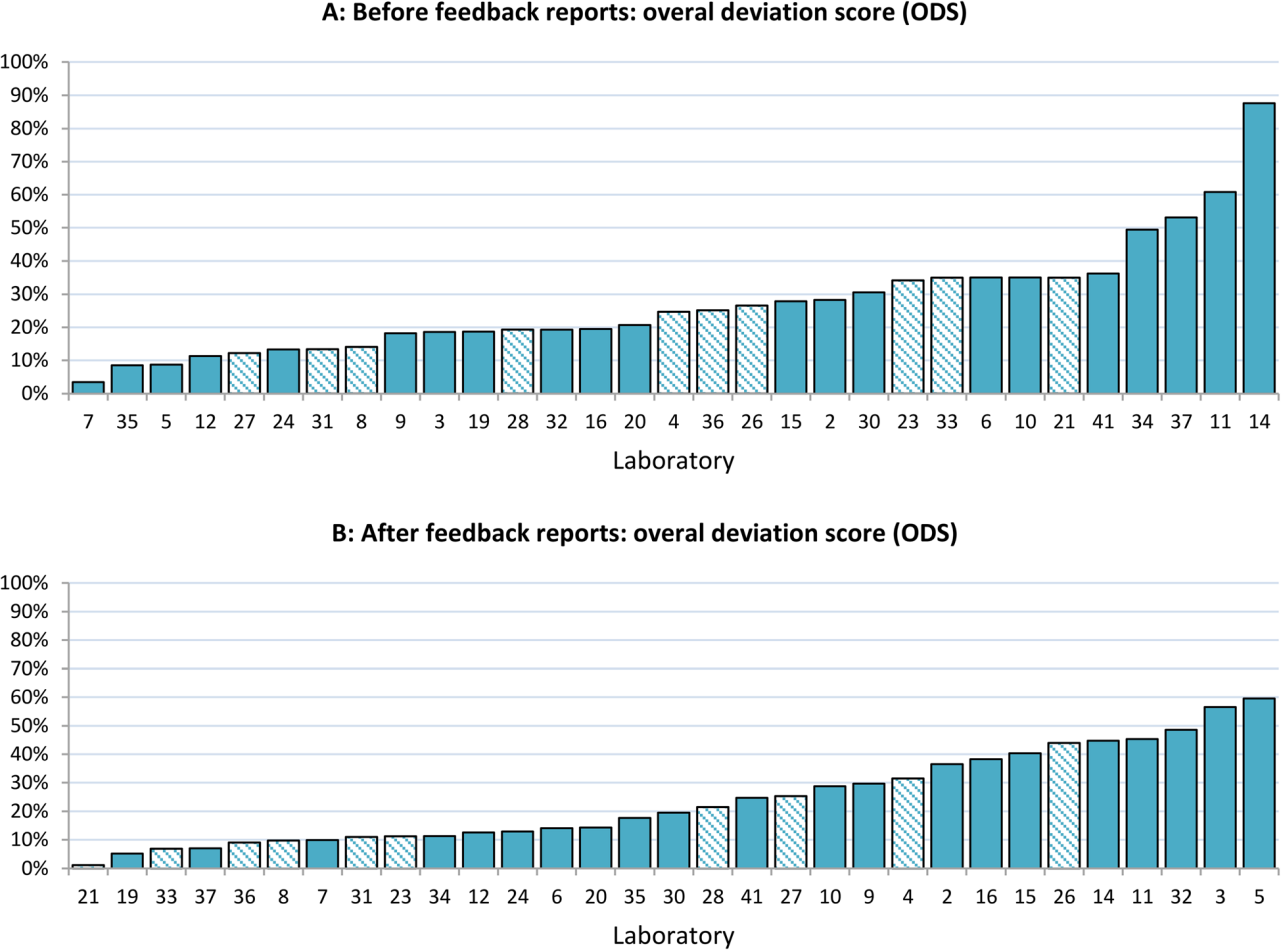
Inter-laboratory (*n* = 31) variation in histologic grading of ductal carcinoma in situ (DCIS) of the breast (**a**) (*n* = 1493) and after feedback reports (**b**) (*n* = 1461). Each bar represents the overall deviation score (ODS) per laboratory. Laboratory numbers for A & B correspond. All laboratories are ranged from lower to higher ODS. The maximum ODS decreased from 87.7 to 59.6% after feedback. Striped bars indicate laboratories who received feedback on pathologist’ level (*n* = 10)

After feedback, the maximum ODS decreased considerably from 87.7 to 59.6%, while the mean ODS also decreased from 27.2 to 24.1% (Fig. 2). The ODS of the individual laboratories did not differ significantly (Wilcoxon signed rank test, *p* = 0.456).

The majority of laboratories became less deviant after feedback for grades II and III (18 laboratories (58.1%) and 17 laboratories (54.8%) respectively), while 11 laboratories (35.5%) became more deviant for both grades. For grade I, the number of laboratories that became less deviant was similar to the number of laboratories that became more deviant; 11 (35.5%) versus 12 (38.8%) laboratories, while 8 (25.8%) laboratories showed a shift of ≤2% (Table 2).

**Table 2:** Type of change in the laboratories after feedback per histologic grade

Type of change	Totalnumber of laboratories (***n*** = 31)	Laboratories with feedback on pathologist’ level (***n*** = 10)	Laboratories without feedback on pathologist’ level (***n*** = 21)	***p***-value
**Grade I**				
**No shift (≤2%)**	8 (25.8%)	1 (10.0%)	7 (33.3%)	0.165*
**Shift(> 2%)**				
Less deviant	11 (35.5%)	6 (60.0%)	5 (23.8%)	0.147**
More deviant	12 (38.7%)	3 (30.0%)	9 (42.9%)	
**Grade II**				
**No shift (≤2%)**	2 (6.5%)	1 (10.0%)	1 (4.8%)	0.579*
**Shift (> 2%)**				
Less deviant	18 (58.1%)	6 (60.0%)	12 (57.1%)	0.732**
More deviant	11 (35.5%)	3 (30.0%)	8 (38.1%)	
**Grade III**				
**No shift (≤2%)**	3 (9.7%)	2 (20.0%)	1 (4.8%)	0.180*
**Shift (> 2%)**				
Less deviant	17 (54.8%)	6 (60.0%)	11 (52.4%)	0.328**
More deviant	11 (35.5%)	2 (20.0%)	9 (42.9%)	

* *p*-value for shift vs. no shift between laboratories with and without feedback on pathologist’ level

** *p*-value for type of change when laboratories shifted > 2% after feedback between laboratories with and without feedback on pathologist’ level

**Fig. 3:**
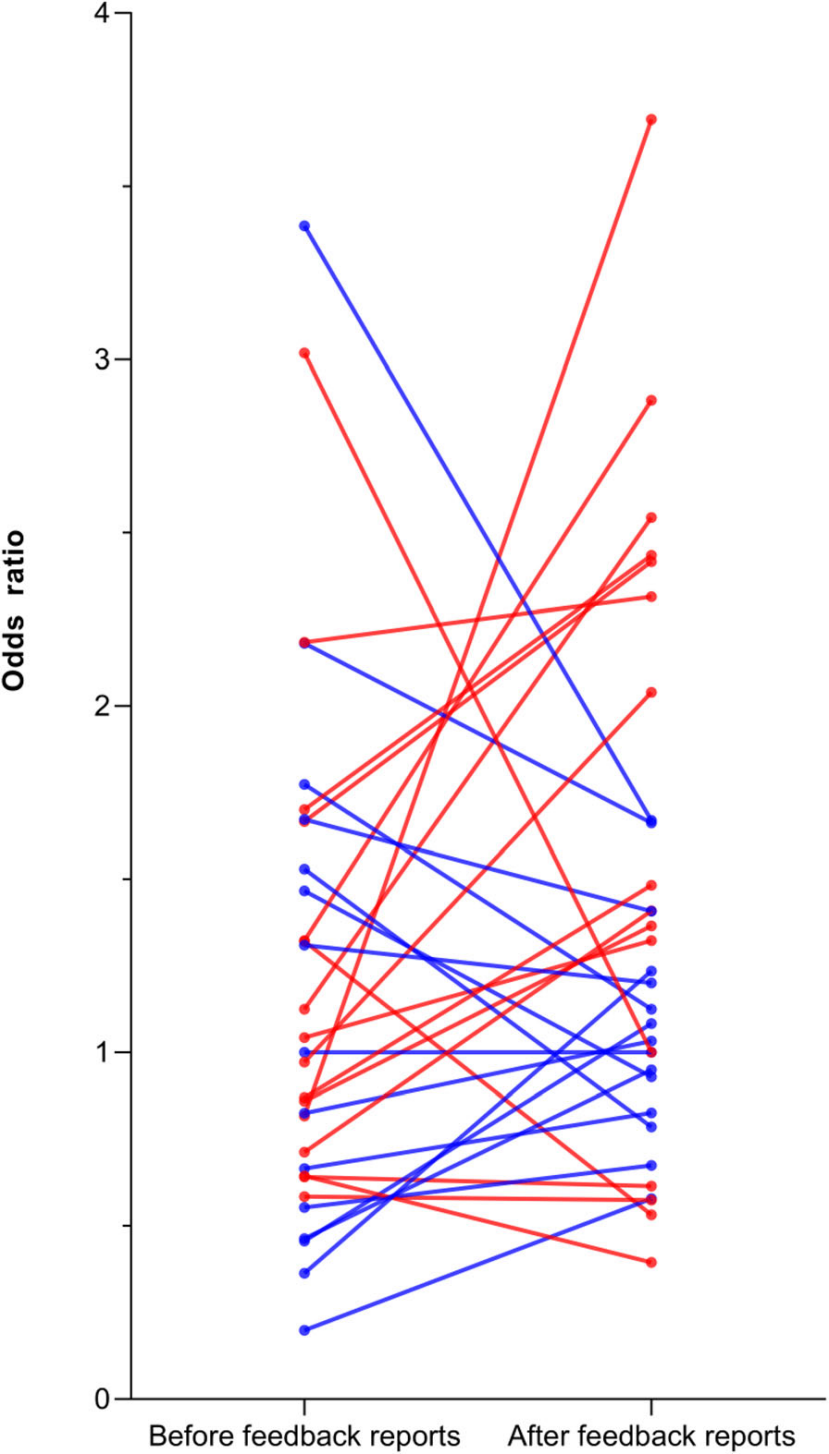
Case-mix adjusted odds ratios per laboratory before and after the feedback reports were calculated by multivariate logistic regression analyses for grade III versus grade I-II ductal carcinoma in situ (DCIS). ORs are adjusted for tumor size and type of surgery. Each laboratory is represented by two dots (before and after feedback) connected by one line. The color of the line and dots shows whether the OR after feedback shifted towards an OR of 1.00, and thus became less deviant (blue) or the OR after feedback shifted away from an OR of 1.00, and thus became more deviant (red) from the reference laboratory

Laboratory 19 was chosen as reference laboratory in the multivariate logistic regression model, as it best resembled the national distribution for grade III versus grade I-II (mean deviation for grade III before and after feedback 1.1%). Laboratory-specific ORs ranged from 0.20 (95% CI: 0.06–0.65) to 3.39 (95% CI: 1.34–8.57) before feedback, resulting in an overall OR range of 3.19. After feedback, laboratory-specific ORs ranged from 0.39 (95% CI: 0.18–0.86) to 3.69 (95% CI: 1.30–10.51), with a corresponding OR range of 3.30 (Fig. 3). Subsequently, the overall OR range increased by 3.5% after feedback. Positive OR-differences within the laboratories did not significantly differ before and after feedback (Wilcoxon signed-rank test, *p* = 0.886).

### Feedback on pathologist’ level

Compared to the slight decrease in mean ODS (from 28.8 to 27.5%) of laboratories who only received feedback on laboratory-level, the mean ODS of the ten laboratories who did also receive feedback on pathologist’ level, showed a larger decrease from 24.0 to 17.1%. As for the type of change in laboratories, it seems that laboratories who also received feedback on pathologist’ level showed more improvement, however, these differences were not statistically significant (Table 2).

## Discussion

Using data from structured pathology reports, we investigated the effect of feedback reports on nationwide inter-laboratory grading variation of DCIS. The decrease in absolute variation for grades II (6.5%) and III (22.0%), as well as the overall majority of laboratories becoming less deviant after feedback, and the decrease of both the mean and maximum ODS seem to indicate a promising decrease in DCIS grading variation. However, absolute variation increased for grade I (6.2%), and the range of case-mix adjusted ORs remained fairly stable and large after feedback. Furthermore, the absolute range between laboratories remains substantial, and maybe even clinically unacceptable, for all grades.

We hypothesize that the lack of consensus on a grading classification [13, 20], which is also reflected by the use of different classifications by the trials [11, 12, 14–16], may be the explanation for these mixed results as we believe that uniform grading starts with the use of single grading classification by all pathologists. Furthermore, in comparison to grading of invasive breast cancer, which is performed according to the modified Bloom and Richardson grading classification (Elston-Ellis modification) [33, 34], with scoring of the three subcategories (tubular differentiation, nuclear polymorphism and mitotic count) grading of DCIS is less standardized. Recently, dichotomous histopathological assessment of ductal carcinoma was studied as an alternative with (better) acceptable degrees of interobserver variability, however, interobserver variation remained considerable and at best acceptable [35]. In addition, other recent data showed that even among 35 expert breast pathologists, the threshold for comedonecrosis is highly variable [36]. This highlights the complexity of histologic grading of DCIS and the need for clear and uniform guidelines [36]. This not only important to the possible implementation of trials results into daily clinical practice, but it may also benefit the quality of the trials itself as central pathology review is not always carried out [37].

Interestingly, 7 out of 38 laboratories that used the synoptic PALGA protocol, were excluded for grading less than 15 DCIS resection specimens (via the protocol), which practically means they grade little over one (pure) DCIS specimen per month. For two laboratories this was because they likely only started using the protocol while another laboratory, stopped using the protocol for unknown reasons. Yet, for the remaining four laboratories, numbers per year (pre- or post-feedback) were fairly stable and low. We would like to emphasize, however, that pathologists within these laboratories may still report DCIS resection specimens outside the protocol, and therefore, they may grade more DCIS than our data would suggest. Nevertheless, if these are the actual numbers of DCIS’ that are assessed in specific pathology laboratories per year, it may be questioned whether this is desirable, especially with regard to the complexity of histologic grading.

Easy data extraction on a large (nationwide) scale and an increased overall completeness of reports [38] were the main reasons to only include synoptic pathology reports. Furthermore, over 80% of breast resection specimens is reported via the synoptic protocol [39].

The mean numbers per histologic grade in this study (13.0% for grade I, 39.6% for grade II and 47.4% for grade III) are in line with other studies [20, 40, 41]. Interestingly, we observed a significant change in grade distribution after feedback as the proportion of grade I tumors increased by over 3%, while the proportion of grade III tumors decreased by a similar percentage. Whether this is initiated by the feedback reports or whether it reflects a true change in the population of DCIS patients remains unknown. Nonetheless, it did make it more difficult to interpret the results regarding deviations from the mean.

Overall, after analyzing the data in an absolute and relative manner, we did observe promising and positive changes, reflected by the decrease in absolute variation for DCIS grade II and III and the decrease of both the mean and maximum ODS. Furthermore, the majority of laboratories became less deviant after feedback for grades II and III. Hence, for grades II and III most deviant laboratories became less deviant, indicating that there is a decrease of the extremes, while changes of individual laboratories (ODS, positive OR-differences) were not significant. In contrast, the results for DCIS grade I showed an increase in the absolute range between laboratories, while the overall range of ORs remained stable and substantially large, ranging from 0.39 (95% CI: 0.18–0.86) to 3.69 (95% CI: 1.30–10.51). This shows that variation in histologic grading is still far from clinically acceptable levels.

Our results confirmed that feedback on the individual level (i.e. the pathologist) may be more effective than feedback on the general level (i.e. laboratories) [42–45]. We observed a larger absolute decrease of the mean ODS of laboratories who also received individual feedback. Furthermore, these laboratories also showed more improvement as compared to laboratories who only received feedback on laboratory-level. Due to the low number of laboratories who received pathologist-specific feedback reports, differences were not statistically significant.

The observed effects on grading variation may not exclusively be attributed to the feedback reports. However, we would like to emphasize that between 2016 and March 1, 2019, besides the feedback reports, no other interventions or guideline changes took place. Furthermore, our previous paper [20] was only first published after feedback reports were sent to the individual pathology laboratories. We believe that these feedback reports may be a useful tool to, at least, monitor grading variation in daily clinical practice.

## Conclusion

We observed a promising decrease in grading variation for DCIS grades II and III, while this was not observed for DCIS grade I. The overall variation for all grades remains substantial, and it seems that histologic grade is far from being a clinically acceptable biomarker for DCIS, let alone be the single biomarker that decides whether patients may be treated. In light of the current ongoing trials, improvement and standardization of DCIS grading is adamant. Continuing with feedback reports, especially on pathologist’ level, helps to create awareness and may open the discussion about nationwide consensus on a single grading classification. In addition, adequate training of (expert breast) pathologists, according to a single classification system, for example by e-learning, may help to establish uniform grading in clinical practice over time.

## Supplementary information

**Additional file 1 Supplementary Fig. 1.** Flowchart of included pathology reports of ductal carcinoma in situ of the breast (DCIS) resection specimens to assess the effect of feedback reports on variation in histologic grading of DCIS.

## Data Availability

All data are available upon request from PALGA (the nationwide registry of histo- and cytopathology in the Netherlands).

## Abbreviations

DCIS: Ductal carcinoma in situ of the breast (DCIS)
PALGA: The nationwide Dutch pathology registry
GDPR: General Data Protection Regulation Act
SKMS: Quality Foundation of the Dutch Association of Medical Specialists
