# Supplementary material for: Grading variation in 2,934 patients with ductal carcinoma in situ of the breast: the effect of laboratory- and pathologist-specific feedback reports
Source: Diagn Pathol. 2020 May 11;15:52. doi: 10.1186/s13000-020-00970-8 (PMC7216330; doi:10.1186/s13000-020-00970-8)
Supplement: Supplementary file 1 — Additional file 1 Supplementary Fig. 1. Flowchart of included pathology reports of ductal carcinoma in situ of the breast (DCIS) resection specimens to assess the effect of feedback reports on variation in histologic grading of DCIS. [file 13000_2020_970_MOESM1_ESM.pdf]

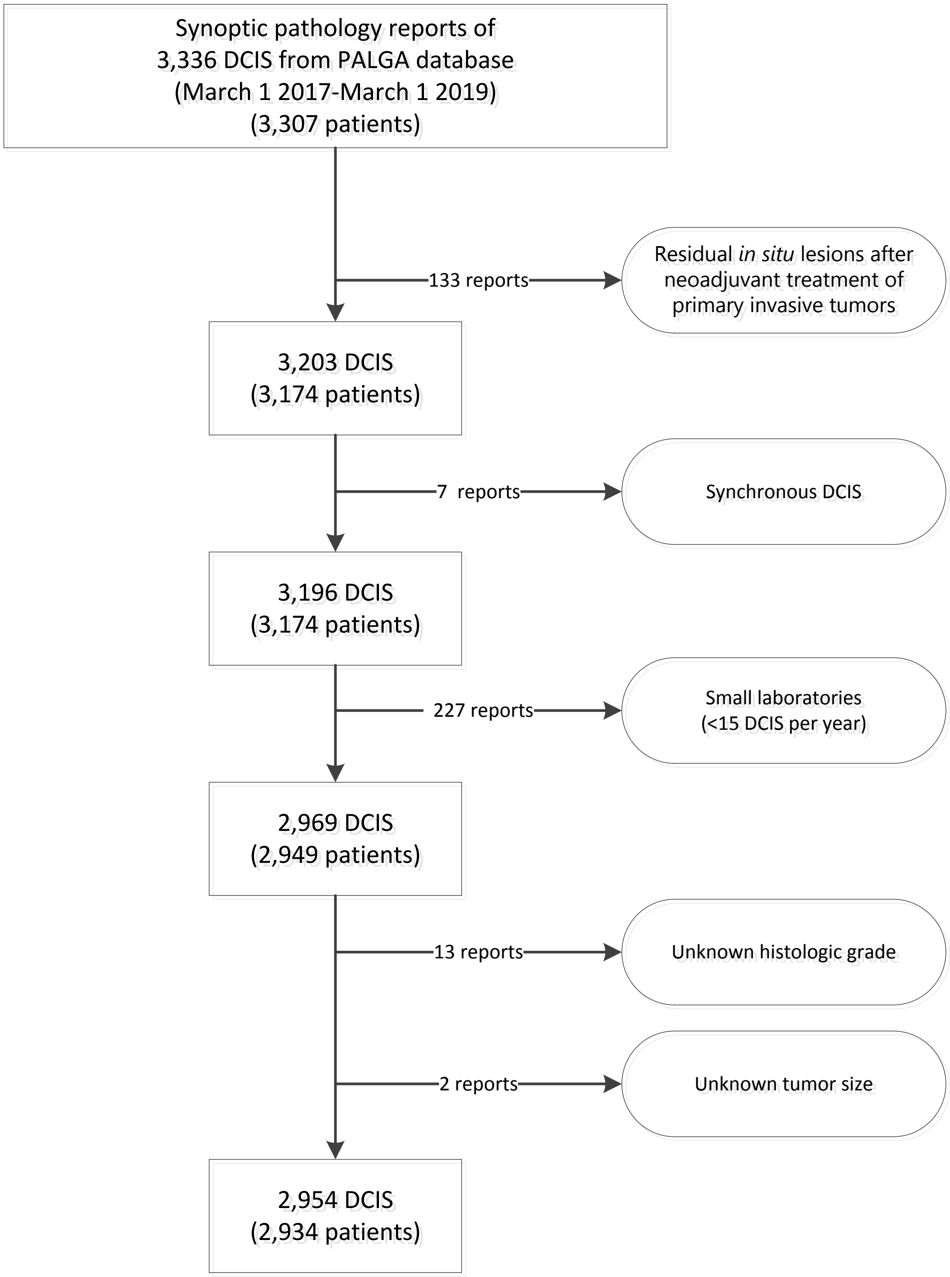

Supplementary figure 1. Flowchart of included pathology reports of ductal carcinoma *in situ* of the breast (DCIS) resection specimens to assess the effect of feedback reports on variation in histologic grading of DCIS.
